# The Use of Statins in Parkinson's and Alzheimer's Disease: A 2021–2025 State‐of‐the‐Art Review of Clinical and Preclinical Evidence

**DOI:** 10.1002/prp2.70280

**Published:** 2026-06-07

**Authors:** Luigi Maria Chiatto, Lucia Buccarello, Giuseppe Carota, Rocco Salvatore Calabrò, Carmela Rifici, Emanuela Esposito, Alessio Ardizzone, Giuseppe Caruso

**Affiliations:** ^1^ Independent Researcher Messina Italy; ^2^ Departmental Faculty of Medicine UniCamillus‐Saint Camillus International University of Health Sciences Rome Italy; ^3^ IRCCS San Camillo Hospital Venice Italy; ^4^ Department of Biomedical and Biotechnological Sciences University of Catania Catania Italy; ^5^ IRCCS Centro Neurolesi Bonino‐Pulejo Messina Italy; ^6^ Department of Chemical, Biological, Pharmaceutical and Environmental Sciences University of Messina Messina Italy

**Keywords:** Alzheimer's disease, disease‐modifying therapies, drug repurposing, neurodegeneration, neuroinflammation, neuroprotection, Parkinson's disease, statins

## Abstract

Statins, widely prescribed for cardiovascular prevention, have emerged as potential disease‐modifying agents in neurodegenerative disorders due to their pleiotropic effects on cholesterol metabolism, neuroinflammation, oxidative stress, and protein aggregation. Over the past decade, growing interest has focused on the potential repurposing of statins for Parkinson's disease (PD) and Alzheimer's disease (AD); however, clinical evidence remains heterogeneous and, in some cases, contradictory. This state‐of‐the‐art review synthesizes clinical and preclinical studies published between 2021 and 2025 to critically evaluate the therapeutic potential and limitations of statins in PD and AD. Recent observational studies and large‐scale cohort analyses suggest that long‐term statin use may be associated with a reduced risk of incident PD and AD, as well as slower cognitive decline in selected patients' subgroups. However, these associations appear to depend on factors such as statin lipophilicity, treatment duration, and genetic background. Preclinical models provide mechanistic support, showing that statins can attenuate neuroinflammation, modulate microglial activation, reduce α‐synuclein aggregation in PD models, and interfere with amyloid‐β production and tau phosphorylation in AD models. Nevertheless, randomized controlled trials remain limited in number and often underpowered, and some reports indicate neutral or even adverse neurological outcomes, underscoring the complexity of cholesterol‐dependent and cholesterol‐independent mechanisms in the central nervous system (CNS). Collectively, the evidence from 2021 to 2025 highlights both the therapeutic promise and the unresolved challenges of statin repurposing in neurodegenerative diseases. Future research should prioritize well‐designed clinical trials and biomarker‐driven patient stratification to determine whether statins can be effectively leveraged as adjunctive disease‐modifying therapies in PD and AD.

## 1 Introduction

Parkinson's disease (PD) and Alzheimer's disease (AD) represent the two most prevalent neurodegenerative disorders worldwide and constitute a rapidly escalating public health challenge in aging societies [1]. Together, they account for a substantial proportion of dementia and movement disorder cases, with profound medical, social, and economic consequences [1]. Despite decades of extensive research, disease‐modifying therapies capable of altering the natural course of PD and AD remain unavailable. Current pharmacological interventions are largely symptomatic, providing transient relief of motor or cognitive manifestations without significantly slowing neurodegeneration [2]. This therapeutic gap has prompted renewed interest in drug repurposing strategies, which aim to leverage the known safety profiles and pharmacokinetic properties of approved compounds to accelerate the identification of candidate disease‐modifying interventions for neurodegenerative disorders.

Among potential drug repurposing strategies, statins have garnered significant interest. These agents were originally developed as inhibitors of 3‐hydroxy‐3‐methylglutaryl–coenzyme A (HMG‐CoA) reductase for the management of hypercholesterolaemia and the prevention of cardiovascular disease and are currently among the most widely prescribed medications worldwide [3]. Beyond lipid‐lowering effects, statins exert pleiotropic actions that include modulation of inflammatory signaling, oxidative stress pathways, endothelial function, and immune cell activation [4]. These cholesterol‐independent effects have positioned statins as potential modulators of pathogenic processes implicated in neurodegeneration [5]. In the CNS, cholesterol homeostasis is tightly regulated and plays a critical role in synaptic integrity, membrane organization, and myelin maintenance [6]. Dysregulation of lipid metabolism, together with chronic neuroinflammation and aberrant protein aggregation, has been increasingly recognized as a convergent pathological axis in both PD and AD, raising the possibility that statins may influence disease‐relevant pathways beyond their established cardiovascular indications [7].

In particular, statins may indirectly affect neurodegenerative trajectories by improving cerebrovascular function and reducing vascular risk factors, which are increasingly appreciated as contributors to cognitive decline and mixed dementia phenotypes [8]. However, the relevance of these mechanisms to human disease remains incompletely understood, and concerns persist regarding the potential impact of cholesterol‐lowering in the brain, particularly given the essential role of cholesterol in neuronal membranes and synapse formation.

Clinical evidence regarding the association between statin use and neurodegenerative outcomes has been heterogeneous and, in some cases, contradictory [9]. Variability in study design, population characteristics, exposure definitions, and outcome measures likely contributes to these inconsistencies. Furthermore, differences among statin classes, particularly in terms of lipophilicity and blood–brain barrier (BBB) permeability, complicate the interpretation of epidemiological findings. Genetic factors, including apolipoprotein E (APOE) genotype and other lipid‐related variants, may further influence individual responses to statin therapy, adding complexity to the clinical landscape [10].

Therefore, in this comprehensive narrative review, we synthesize and critically appraise preclinical and clinical evidence published between 2021 and 2025 on the use of statins in PD and AD. By integrating mechanistic insights with epidemiological and translational findings, we aim to delineate convergent and divergent lines of evidence, identify key sources of heterogeneity across studies, and highlight unresolved controversies. Determining whether and how statins can be repurposed as adjunctive disease‐modifying therapies in PD and AD may support precision medicine approaches and accelerate the translation of mechanistic insights into clinical applications.

### 1.1 Methodology

This state‐of‐the‐art review was conducted through a literature search performed in PubMed to identify relevant studies published between 2021 and 2025 regarding the potential role of statins in AD and PD.

The search strategy included combinations of the following keywords: “statins”, “Alzheimer's disease”, “Parkinson's disease”, “neurodegeneration”, “neuroprotection”, “dementia”, “amyloid‐beta”, “tau”, “α‐synuclein”, and “inflammation”.

Original research articles published in English were considered eligible, including preclinical studies (in vitro and animal models) and each type of clinical studies. Reviews, editorials, commentaries, and conference abstracts were excluded. Studies were selected based on their relevance to the topic and their contribution to understanding the potential neuroprotective and disease‐modifying effects of statins in AD and PD.

As this work was designed as a narrative state‐of‐the‐art review rather than a systematic review or meta‐analysis, PRISMA guidelines were not formally applied. However, efforts were made to ensure a balanced and updated overview of the currently available evidence.

## 2 Statins: Pharmacological Basis and Relevance to the CNS

Statins constitute a pharmacologically well‐characterized class of small‐molecule inhibitors of 3‐hydroxy‐3‐methylglutaryl–coenzyme A (HMG‐CoA) reductase, the rate‐limiting enzyme of the mevalonate pathway [11]. By competitively inhibiting HMG‐CoA reductase, statins reduce hepatic cholesterol synthesis and upregulate low‐density lipoprotein (LDL) receptor expression, leading to decreased circulating LDL cholesterol levels [12]. Although originally developed for cardiovascular prevention, the pharmacological profile of statins extends beyond lipid lowering, encompassing a range of so‐called pleiotropic effects that are increasingly recognized as potentially relevant to neurodegenerative disorders [13]. These effects arise from the inhibition of downstream isoprenoid intermediates of the mevalonate pathway, including farnesyl pyrophosphate and geranylgeranyl pyrophosphate, which are essential for the posttranslational prenylation of small GTPases such as Ras, Rho, and Rac [14].

From a pharmacokinetic perspective, statins display substantial heterogeneity in lipophilicity, metabolic pathways, and tissue distribution, features that are likely to shape their CNS effects [15]. Lipophilic statins (e.g., simvastatin, atorvastatin, and lovastatin) exhibit higher passive diffusion across cellular membranes and may cross the BBB to a greater extent than hydrophilic statins (such as pravastatin and rosuvastatin), which rely more heavily on active transport mechanisms and display limited CNS penetration under physiological conditions [16]. However, BBB permeability is not static and may be altered by aging, neuroinflammation, and neurodegeneration, potentially influencing statin exposure within the brain across different disease stages. In addition, statins are substrates of multiple transporters (including organic anion transporting polypeptides and P‐glycoprotein) and are primarily metabolized by cytochrome P450 isoenzymes (notably CYP3A4 and CYP2C9 for several lipophilic statins), thereby introducing interindividual variability in systemic and possibly CNS drug levels [17]. These pharmacokinetic determinants underscore the importance of statin class, dose, and treatment duration when interpreting clinical and preclinical findings in PD and AD.

At the cellular level, statins exert anti‐inflammatory and immunomodulatory effects that may be relevant to neurodegeneration [18]. Inhibition of isoprenoid synthesis leads to reduced prenylation of Rho family GTPases, resulting in altered cytoskeletal dynamics and attenuation of pro‐inflammatory signaling cascades, including nuclear factor‐κB (NF‐κB)–dependent transcription [19]. In microglia and astrocytes, statins have been shown to shift activation states toward less pro‐inflammatory phenotypes in experimental settings, with downstream effects on cytokine release, nitric oxide production, and reactive oxygen species (ROS) generation [20]. Given the central role of chronic, low‐grade neuroinflammation in PD and AD pathogenesis, as well as the contribution of nitric oxide and ROS overproduced by immune cells to pro‐inflammatory maintenance and progression [21, 22], these pharmacodynamic actions provide a mechanistic framework for potential disease‐modifying effects. Statins have also been reported to modulate endothelial nitric oxide synthase activity and improve cerebrovascular function, which may indirectly influence neuronal resilience by enhancing cerebral perfusion and reducing vascular contributions to cognitive impairment and dementia [23].

However, these effects could be context dependent and may vary according to statin concentration, exposure duration, and cellular model, raising important questions regarding dose–response relationships and translational relevance. Notwithstanding these potentially beneficial mechanisms, the pharmacological modulation of cholesterol metabolism in the CNS raises safety considerations.

In addition, drug–drug interactions and patient‐specific factors further complicate the pharmacological landscape of statin repurposing in PD and AD [24]. Polypharmacy is prevalent among elderly populations and patients with neurodegenerative diseases, increasing the risk of pharmacokinetic interactions mediated by cytochrome P450 enzymes or transporter systems [24]. Moreover, genetic variability in drug‐metabolizing enzymes and lipid‐related pathways may influence both systemic exposure and CNS effects of statins [25]. Collectively, the pharmacological properties of statins provide a compelling, yet complex, framework for their potential repositioning in PD and AD. Thus, a comprehensive understanding of statin pharmacology in the context of the aging and diseased brain will be essential to determine whether specific statins, dosing regimens, and treatment windows can be rationally leveraged to achieve disease‐modifying effects in PD and AD.

## 3 The Pathophysiology and Clinical Features of AD

AD is the most common form of dementia, accounting for approximately 60%–80% of cases [26]. The pathology is a progressive neurodegenerative disease that primarily affects older adults, but early‐onset forms of this disease can occur [27]. AD is characterized by a progressive and often rapid decline in cognitive abilities, including memory, reasoning, and behavior [28]. This deterioration extensively impairs daily functioning, eventually preventing individuals from performing routine activities independently and resulting in a marked reduction in quality of life [29]. According to the 2019 World Alzheimer Reporter, an estimated 50 million people worldwide are currently living with AD, and this number is expected to triple by 2050 [30].

From a psychological perspective, significant critical issues emerge: one of the most significant concerns is communicating the diagnosis to patients and their families. It is important not to rush disclosure, as the knowledge of suffering from an incurable disease can lead to a secondary worsening of symptoms after the diagnosis is made. Symptoms can appear at different times from the diagnosis: it has been reported that depression, mood swings, social isolation, and suicidal ideation are very common up to 2 years before diagnosis; as well, patients develop hallucinations, paranoia, and delusions within a month of receiving the diagnosis; furthermore, irritability, agitation, and aggressive behavior could appear within the first year following diagnosis [31].

**FIGURE 1:**
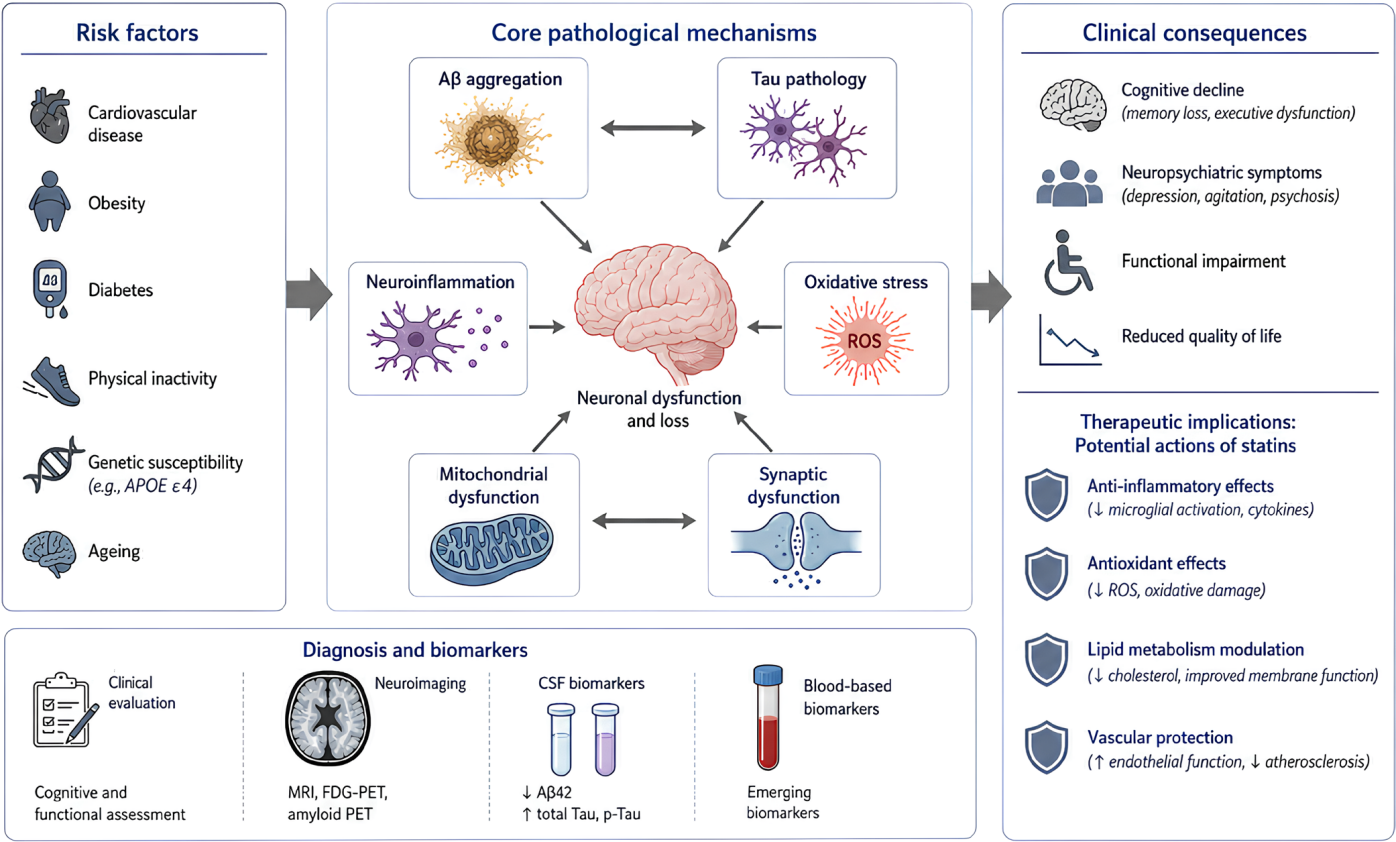
Illustration of neuropathological hallmarks of AD.

AD places an enormous emotional, physical, and financial burden also on families, often requiring long‐term support. The economic costs associated with AD are substantial and include healthcare expenses, lost productivity, and social support services [32]. It is therefore essential that healthcare providers have access to clinical tools that can help them implement effective therapeutic interventions. Pathogenically, AD is characterized by the accumulation of amyloid‐β (Aβ) plaques and neurofibrillary tangles (NFTs) [33], including regions related to cognition [34]. Other mechanisms, such as neuroinflammation, mitochondrial dysfunction, and synaptic dysfunction, have been implicated in its pathogenesis [35]. Furthermore, cardiovascular disease, obesity, diabetes, and specific lifestyle factors increase the likelihood of developing AD [36]. Diagnosis is established through a combination of cognitive and behavioral tests, blood and cerebrospinal fluid analyses, neurological examinations, and brain imaging techniques. Aβ and tau protein levels in the cerebrospinal fluid are noninvasive or minimally invasive biomarkers for early diagnosis [37]. Several radiolabeled tracers are currently under development for neuroimaging applications, enabling the investigation of key pathological processes such as cerebral hypoperfusion, hypometabolism, neuroinflammation, and Aβ accumulation [38]. Currently available therapies for AD are primarily focused on symptom management and mitigation but, unfortunately, they do not provide a curative or fully disease‐remitting treatment for affected patients. Therefore, growing attention has been directed toward the identification of disease‐modifying strategies, including the repurposing of statins, which may target multiple pathways implicated in AD neurodegeneration. All AD features are summarized in Figure 1.

### 3.1 Preclinical Studies on Statins and Cognitive Decline in AD From 2021 to 2025

Preclinical studies provide mechanistic support for the potential neuroprotective effects of statins in AD models through modulation of multiple pathways implicated in neurodegeneration. Beyond cholesterol lowering, statins exert pleiotropic effects by inhibiting HMG‐CoA reductase and downstream isoprenoid synthesis, thereby modulating small GTPase signaling and reducing Aβ production and aggregation. They also attenuate oxidative stress via inhibition of NADPH oxidase and activation of the Nrf2/HO‐1 pathway and suppress neuroinflammation through inhibition of NF‐κB signaling and downstream cytokines such as TNF‐α, IL‐1β, and IL‐6. In addition, statins enhance synaptic plasticity and cognitive function by modulating key pathways involved in neuronal survival and long‐term potentiation, including GSK‐3β inhibition and CREB activation [39].

In an early AD mouse model as APP transgenic mice, simvastatin treatment improved memory performance and promoted maturation of dentate gyrus granule neurons by restoring dendritic complexity, an effect linked to activation of the Wnt/β‐catenin signaling pathway through downregulation of the Wnt inhibitor DKK1 and increased expression of the downstream target Prox1 [40]. Consistently, in experimental models of neurodegeneration, statin treatment has been shown to attenuate learning and memory deficits while reducing oxidative stress, inflammation, and apoptotic signaling. These effects are accompanied by decreased acetylcholinesterase activity and AD‐related markers such as presenilin‐1 and hyperphosphorylated tau [41]. These neuroprotective effects were associated with modulation of several signaling cascades involved in neuronal survival and plasticity, including Wnt/β‐catenin, ERK, and Akt pathways, as well as reduced astrocytic and microglial activation [40].

Additional mechanistic evidence reported by Langness et al. [42] indicates that statins may directly interfere with amyloidogenic pathways. In human neuronal models, cholesterol‐lowering drugs were shown to reduce Aβ generation by altering APP processing, decreasing the interaction between APP and BACE1 while promoting APP dimerization, thereby limiting β‐secretase–mediated cleavage [42]. Similarly, in vitro studies performed by Nedaei et al. [43] demonstrated that the calcium‐free form of atorvastatin can directly inhibit Aβ₁₋42 aggregation by binding to the hydrophobic KLVF region of the peptide and preventing its transition toward β‐sheet–rich fibrillar conformations. Cholesterol metabolism has also been implicated in ApoE‐related pathogenic signaling, as ApoE4 and the cholesterol metabolite 27‐hydroxycholesterol were shown to activate the C/EBPβ/δ‐secretase pathway, promoting AD‐related pathology. Notably, inhibition of cholesterol synthesis with lovastatin attenuated these effects, further supporting the link between cholesterol homeostasis and ApoE4‐driven neurodegeneration [44].

Complementary drug‐repurposing approaches further identified lovastatin as a candidate compound capable of reducing Aβ production in cellular and in vivo models through modulation of amyloidogenic enzymes such as BACE1 and APH1α as proposed by Liu et al. [45]. Beyond direct effects on amyloid processing, statins may exert broader neuroprotective actions through transcriptional and inflammatory regulation. Indeed, statins have been shown to modulate the activity of the nuclear receptor Nurr1, resulting in suppression of astrocyte‐mediated inflammatory responses and induction of neuroprotective gene expression programs in neuronal cells [46].

In vivo studies also suggest that statins may ameliorate cognitive impairment associated with metabolic and vascular risk factors. In a mouse model of hyperhomocysteinemia‐induced cognitive dysfunction, atorvastatin improved recognition memory and reversed glial gene expression alterations, potentially through reductions in circulating homocysteine rather than changes in cholesterol levels [47].

Additional studies suggest that statins may modulate systemic mechanisms contributing to neurodegeneration. In an ovariectomized/D‐galactose rat model of AD, simvastatin improved learning and memory performance while reducing hippocampal amyloid deposition, neuronal loss, and levels of inflammatory cytokines such as TNF‐α and IL‐1β. These effects were accompanied by modulation of the gut microbiota and increased production of short‐chain fatty acids, suggesting involvement of the gut–brain axis in the observed neuroprotective effects [48]. Experimental studies performed by Atef et al. [49] using aluminum chloride‐induced models of AD further demonstrated that simvastatin improves cognitive deficits and reduces amyloid burden while modulating signaling pathways involved in neuroinflammation and neuronal survival. Specifically, these effects were associated with activation of the TGF‐β1/SMAD2 and β‐catenin pathways, along with inhibition of GSK3β and TLR4 signaling [49]. In fact, both TLRs and TGF‐β1 represent pharmacological targets the modulation of which has been often exploited to counteract pro‐inflammatory phenomena [50, 51]. Moreover, strategies aimed at enhancing drug delivery have shown that simvastatin‐loaded lysosome‐targeting nano‐chimeras can promote the degradation of the receptor for advanced glycation end products (RAGE), a key mediator of AD pathology. This approach resulted in attenuation of disease‐related features both in vitro and in vivo [52].

Nanoparticle‐based formulations of statins have also demonstrated beneficial effects on neuronal plasticity and neurogenesis, as rosuvastatin coated with nanochitosan reduced hippocampal amyloid deposition, increased neuronal marker expression, and improved learning and memory performance in Aβ‐induced rat models [53]. Additional evidence also indicates that statins may enhance Aβ clearance and neuronal resilience in vivo.

In a streptozotocin‐induced rat model of AD, combined treatment with memantine and rosuvastatin improved cognitive performance and attenuated hippocampal injury while suppressing the TGF‐β1/Smad2/p21 signaling pathway and enhancing transporter systems involved in Aβ efflux across the blood–brain barrier, including ABCA1 and LRP1 [54]. Evidence from additional experimental models further suggests potential synergistic therapeutic interactions. In an aluminum chloride‐induced rat model of AD, atorvastatin improved cognitive performance, reduced oxidative stress, and inhibited acetylcholinesterase activity, with even greater neuroprotective effects observed when combined with magnesium L‐threonate [55]. Similarly, multi‐omic analyses in transgenic tauopathy mice and human subjects indicated that statin therapy can influence tau‐related pathways, as atorvastatin reduced phosphorylated tau levels and showed additive effects when combined with the angiotensin‐converting enzyme inhibitor lisinopril [56].

Furthermore, emerging evidence suggests that statins may contribute to disease‐modifying effects within multidrug therapeutic strategies. In AppNL‐G‐F knock‐in mice, a polypharmacy regimen including simvastatin improved memory performance, reduced amyloid burden and neuroinflammation, and modulated AD‐associated metabolomic profiles in male mice, highlighting potential synergistic drug interactions and marked sex‐specific responses [57]. All preclinical studies related to the use of statins for the treatment of AD are summarized in Table 1.

**TABLE 1:** Summary of preclinical studies evaluating statins as a pharmacological intervention in AD from 2021 to 2025.

First author and year of publication	Intervention	Key findings–related pathway	Effect on AD	References
Tong et al.; 2022	This preclinical study tested whether simvastatin could improve memory and enhance neurogenesis in AD disease mouse model by using APP mice. The authors focused on the Wnt/β‐catenin signaling pathway, a key regulator of neuronal growth and maturation, as a potential mechanism underlying these effects.	In an AD mouse model, simvastatin improved memory, particularly spatial memory, by promoting hippocampal neuron maturation. This effect was linked to activation of the Wnt/β‐catenin signaling pathway rather than to a reduction in amyloid plaques.	Simvastatin does not cure AD, but it attenuates cognitive deficits and supports neuronal function in mouse models, suggesting a possible neuroprotective and symptomatic role, which needs to be further verified in humans.	[40]
Salari et al.; 2024	This experimental study evaluated the neuroprotective effects of simvastatin in a TMT‐induced animal model of neurodegeneration with AD‐like features.	The study examined mechanisms involved in neurodegeneration, including brain inflammation, oxidative stress, and apoptosis. Simvastatin treatment reduced inflammatory and oxidative damage, normalized AD‐related molecular markers, and improved learning and memory in treated rodents compared with controls.	Simvastatin attenuated TMT‐induced neurotoxicity by reducing inflammation and oxidative stress, improving cognition, and modulating neurodegeneration‐related markers.	[41]
Eroli et al.; 2025	The study assessed how commonly used drugs and their combinations affect AD‐related changes in male and female App^NL‐G‐F^ knock‐in mice, also considering sex‐specific effects.	Drug combinations affect amyloid‐tau, inflammatory, metabolic, and neuroplasticity pathways, with sex‐dependent effects, suggesting that simultaneous modulation of multiple biological pathways may have therapeutic potential for AD.	A combination of metoprolol, simvastatin, aspirin, paracetamol, and citalopram improved memory, reduced amyloid burden and neuroinflammation, and modulated AD‐associated metabolomic signatures in male mice, with negligible effects in female mice.	[57]
Zidan et al.; 2024	This preclinical study used a rat model of AD induced by intracerebroventricular streptozotocin (ICV‐STZ). The researchers evaluated a 28‐day combination therapy with memantine (an NMDA receptor antagonist used in AD) and rosuvastatin, investigating their effects on memory, cognitive function, and hippocampal injury.	The study showed that treatment improved cognition by modulating TGF‐β1/Smad signaling and promoting Aβ clearance via BBB transporters and related microRNAs.	The combination of memantine and rosuvastatin reduced memory deficits and hippocampal neurodegeneration in the AD rat model. It also modulated the TGF‐β1/Smad signaling pathway, improved Aβ clearance, and regulated microRNAs related to blood–brain barrier function and amyloid metabolism.	[54]
Liu et al.; 2025	This experimental study explored drug repurposing strategies for AD by targeting proteins that interact with lactoferrin. Lactoferrin is an immune‐related glycoprotein involved in iron transport, inflammation, and immune modulation, and its interacting proteins may represent potential therapeutic targets.	The study suggests that targeting lactoferrin‐related pathways may reduce neuroinflammation and pathogenic proteins accumulation in AD.	The study identified existing compounds that may modulate lactoferrin‐related pathways, potentially affecting AD pathology, inflammation, and Aβ/tau clearance.	[45]
Nedaei et al.; 2022	The study examined whether calcium‐free atorvastatin modulates Aβ1–42 aggregation in vitro using structural and aggregation assays.	The study showed that calcium‐free atorvastatin inhibits Aβ1–42 aggregation by interacting with K16–V18 residues and reducing β‐fibril formation.	Ca‐free atorvastatin inhibited Aβ1–42 aggregation in vitro, reducing toxic β‐fibrillar structures and suggesting a potential anti‐amyloid strategy for AD.	[43]
Zahedi et al.; 2023	The study evaluated the effects of simvastatin in an AD rat model, showing improvements in learning and memory. These benefits were linked not only to central neural effects but also to modulation of the gut microbiota, suggesting involvement of the gut–brain axis in the drug's neuroprotective action.	Simvastatin may modulate the gut–brain axis, reducing inflammation and lipid dysregulation while supporting synaptic function and cognition.	The study tested simvastatin in an AD experimental model, showing improvements in cognition and memory. The beneficial effects appear to involve not only central brain mechanisms but also metabolic and immune pathways, particularly modulation of the gut microbiota.	[48]
Wang et al.; 2025	The study developed a nanochimera (endoTAC) designed to promote the degradation of pathological proteins through the endocytosis–lysosomal pathway. The system targets the RAGE receptor, which is implicated in AD by facilitating the entry of toxic molecules into the brain and promoting inflammatory responses.	The strategy promotes lysosome‐mediated degradation of target proteins through the endocytic pathway, a targeted protein‐degradation approach. The nanochimera binds the RAGE receptor, enhancing its internalization and lysosomal degradation, which may reduce BBB dysfunction and the accumulation of toxic molecules in AD models.	endoTAC showed high affinity for RAGE and promoted greater degradation of this receptor compared with non‐multivalent systems. The nanochimera also accumulated efficiently in diseased brain tissue, suggesting penetration across a compromised BBB. When loaded with simvastatin (SV@endoTAC), it reversed pathological features of AD models both in vitro and in vivo.	[52]
Wang et al.; 2021	The study used mice lacking the α7‐nAChR receptor (α7‐KO) to examine Aβ physiological functions. Normally, Aβ interacts with α7‐nAChR to support synaptic plasticity and memory. Deletion of this receptor disrupted this interaction, leading to increased Aβ levels and the development of AD‐like features.	These findings suggest that α7‐nAChR deficiency may contribute to AD progression by promoting amyloid and tau pathology, synaptic dysfunction, neuronal loss, and neuroinflammation.	α7‐nAChR loss may drive AD‐like neurodegeneration by linking amyloid accumulation, tau pathology, synaptic impairment, and neuroinflammatory responses.	[44]
Langness et al.; 2021	In this study, human iPSC‐derived neurons carrying familial or sporadic AD‐related mutations were treated with cholesterol‐lowering drugs, including statins, to investigate how altering cellular cholesterol metabolism affects APP processing and Aβ production.	The study indicates that cholesterol depletion may modulate amyloidogenic APP processing and Aβ generation, highlighting cholesterol homeostasis as a relevant factor in AD pathology.	These findings suggest that cholesterol homeostasis may contribute to AD pathology by modulating APP processing and Aβ generation.	[42]
Weekman et al.; 2023	The study used a mouse model of hyperhomocysteinemia, a condition linked to vascular risk and neurodegeneration relevant to AD. Mice received atorvastatin for 14 weeks to model chronic treatment, while control groups remained untreated on a normal diet.	Atorvastatin reduced homocysteine levels and modulated oligodendrocyte and microglial inflammatory responses, with limited effects on astrocytes and neurovascular markers.	Atorvastatin improved cognitive performance in HHcy mice, as shown by better results in object recognition tests. The treatment also reduced neuroinflammatory responses by normalizing glial gene expression and lowering homocysteine levels, with potential involvement of signaling pathways related to neuronal survival and plasticity (e.g., MAPK/Akt/PI3K).	[47]
Gangoda et al.; 2023	The study evaluated atorvastatin and magnesium L‐threonate, alone or combined, in an AlCl_3_‐induced AD mouse model, assessing cognition, oxidative stress, inflammation, and AD‐related brain proteins.	The treatment acted on multiple AD‐related pathways. It reduced oxidative stress by enhancing neuronal antioxidant defenses and decreased neuroinflammatory markers linked to microglial activation. Increased brain Mg^2+^ from magnesium L‐threonate also supported synaptic plasticity and memory function.	Atorvastatin plus magnesium L‐threonate synergistically improved memory and reduced oxidative stress and neuroinflammation in the AD model.	[55]
Collu et al.; 2023	The study evaluated how clinically used ACE inhibitors and statins modulate AD‐related proteomic and transcriptomic profiles, given the link between hypertension, hyperlipidemia, and dementia risk.	Lisinopril and atorvastatin reduced tau‐related pathology and modulated inflammatory, lipid metabolism, and oxidative stress pathways relevant to AD.	ACE inhibitor and statin therapies modulate molecular pathways associated with AD. Changes in omic profiles and the reduction of pathological forms of tau suggest a potentially protective biological effect against neurodegeneration.	[56]
Atef et al.; 2023	The study evaluated simvastatin as an experimental treatment in a mouse model of AD induced by aluminum chloride (AlCl_3_). After AD‐like pathology was induced for 6 weeks, mice were treated with simvastatin (10 or 20 mg/kg) or donepezil (3 mg/kg) for another 6 weeks to assess cognitive and neurochemical outcomes.	Simvastatin showed neuroprotective effects by enhancing TGF‐β1/SMAD2 and β‐catenin signaling while reducing GSK3β, AChE, and Aβ1‐42 levels.	In the AlCl_3_‐induced AD model, simvastatin improved memory and neurological performance and reduced histopathological brain alterations. These benefits were associated with modulation of TGF‐β1/SMAD2 and GSK3β/β‐catenin signaling pathways and reduced levels of AChE and Aβ1‐42. Overall, the findings indicate a neuroprotective effect with improved cellular survival mechanisms.	[49]
Modiri et al.; 2025	The study used a rat model of AD induced by intracerebral injection of Aβ. Animals were divided into control, AD model, AD + nanochitosan, AD + rosuvastatin, and AD + nanochitosan‐coated rosuvastatin groups. The nanochitosan formulation was designed to enhance rosuvastatin delivery to the brain.	Rosuvastatin, especially with nanochitosan delivery, reduced Aβ plaques and hippocampal neuronal death, while improving neurogenesis‐related markers.	Rosuvastatin, especially with nanochitosan delivery, restored neuronal and neurogenesis markers and improved learning and memory performance.	[53]

### 3.2 Clinical Studies on Statins and Cognitive Decline in AD From 2021 to 2025

Recent large‐scale observational studies provide heterogeneous evidence concerning the association between statin use and cognitive outcomes. A comprehensive meta‐analysis by Westphal Filho et al. [58], including 55 observational studies and over 7.7 million individuals, reported a reduced risk of all‐cause dementia (HR ≈0.86) and AD (HR ≈0.82) associated with statin use, with a more pronounced effect observed following long‐term exposure (> 3 years). However, findings from individual cohort studies remain inconsistent. For instance, in a cohort of 18 846 participants without prior dementia or major cardiovascular events, statin exposure was not associated with the onset of dementia or mild cognitive impairment, nor with significant longitudinal changes in global cognitive performance [8]. Similarly, initiation of statin therapy did not significantly modify the long‐term incidence of AD or related dementias in quasi‐experimental analyses, suggesting that modulation of vascular risk factors alone may be insufficient to prevent clinical AD [59].

Evidence from prodromal stages and at‐risk populations suggests a time‐ and exposure‐dependent association between statin use and AD risk. In a nationwide Korean cohort of over 119 000 adults aged ≥ 60 years, overall statin use was not associated with AD incidence; however, short‐term exposure was linked to an increased risk of AD, whereas long‐term and persistent use, as well as higher cumulative doses, were associated with a reduced risk, indicating a potential dose–duration effect [60].

Initiation of statin therapy after age 65 was associated with a lower risk of clinical AD and slower cognitive decline, with stronger associations observed in APOE ε4 carriers, supporting a possible gene–treatment interaction [61]. Modest protective associations have also been reported in selected subgroups, including older women and individuals with more favorable metabolic profiles [62], as well as in cohorts in which lower LDL cholesterol levels and statin use were associated with reduced dementia risk [63, 64]. Neuroimaging and biomarker studies provide mechanistic context for these epidemiological associations. Longitudinal PET analyses suggest that statin use may be associated with reduced or stabilized age‐related Aβ accumulation in cognitively unimpaired individuals, whereas no effect is observed once MCI or AD is established, indicating that potential benefits may be restricted to preclinical disease stages [65]. Structural MRI data further indicate that statin use is associated with greater cortical thickness across multiple brain regions in healthy controls, individuals with MCI and patients with early AD, and with reduced cortical thinning over time in MCI, consistent with a possible neuroprotective signal at early stages of disease [66]. At the molecular level, statin exposure has been associated with altered Aβ transport in peripheral blood [67] and with changes in the expression of genes and pathways implicated in AD pathogenesis, including lipid metabolism, inflammation and mitochondrial function, providing biological plausibility without direct evidence of clinical efficacy [68]. In patients with established AD, most studies report neutral effects on cognitive trajectories despite potential systemic benefits. Continued statin use in individuals with mild‐to‐moderate AD was not associated with slower cognitive decline or reduced dementia progression, although statins appeared safe and were not linked to increased adverse events [69].

In a large Korean cohort of patients with AD, statin use was associated with reduced all‐cause mortality but did not delay the initiation of memantine, suggesting limited impact on symptomatic disease progression [70]. Heterogeneous associations between statin use and cognition have been reported in clinical databases, with statin‐specific signals for simvastatin and atorvastatin, although causal inference is precluded by the observational nature of these analyses [71, 72, 73]. Not all datasets show a protective association between statin use and AD risk. For example, in individuals with familial hypercholesterolemia, statin exposure was not linked to either a decreased or increased risk of AD or overall dementia, suggesting that cholesterol lowering alone may not influence disease risk in this population [74]. Other large‐scale analyses have reported an increased risk of AD associated with statin use in specific genetic and clinical subgroups, highlighting the potential importance of personalized risk stratification [75]. Consistent with this view, statin use has been associated with a moderate reduction in dementia severity selectively in APOE ε4 carriers, with no significant effects in noncarriers, suggesting that genetic background may critically shape treatment–outcome relationships [76]. Additional observational and translational studies further underscore the complexity of the statin–cognition relationship. Combinations of antihypertensive agents with statins or metformin were associated with a stronger reduction in dementia risk compared with antihypertensive therapy alone, pointing to potential synergistic effects on vascular and metabolic pathways [77]. Changes in cognitive profiles have also been associated with statin and/or coenzyme Q10 use, possibly through modulation of oxidative stress, mitochondrial metabolism and systemic inflammation, although these findings remain preliminary and hypothesis‐generating [78]. In line with a vascular contribution to cognitive decline, interventions targeting endothelial nitric oxide signaling have been associated with increased cerebral blood flow and modest cognitive benefits in patients with MCI or mild AD, reinforcing the relevance of cerebrovascular mechanisms in disease progression [79].

Finally, emerging interventional and biomarker‐driven studies point to potential biological effects of statins in specific clinical contexts. In patients with AD and dyslipidaemia, rosuvastatin treatment has been associated with reduced tau levels and increased miR‐124‐3p expression. These findings are consistent with modulation of neuroinflammatory and neuroprotective pathways, although their clinical significance remains to be established [80]. Together, these findings indicate that statins do not exert a uniform disease‐modifying effect in established AD, but may be associated with modest, context‐dependent biological and clinical signals at preclinical or prodromal stages, with treatment duration, statin class and genetic background emerging as critical modifiers of observed outcomes [8, 59, 60, 61]. All clinical studies related to the use of statin for the treatment of AD are summarized in Table 2.

**TABLE 2:** Summary of clinical studies evaluating statins as a pharmacological intervention in AD from 2021 to 2025.

First author and year of publication	Intervention	Key findings–related pathway	Effect on AD	References
Zhou et al.; 2021	Observational comparison of statin users vs. non‐users within a randomized aspirin trial to assess dementia, AD, MCI, and cognitive decline.	Investigate associations between statin use and cognitive decline and incident dementia among older adults.	Statins have not been shown to have a preventive effect on AD, but they are not found to be harmful to cognitive function either.	[8]
Petek et al.; 2023	Longitudinal cohort comparing cognitive trajectories in statin users vs. non‐users using mixed‐effects models and dose–response analysis.	Association between statin use and cognitive decline in AD/mixed dementia; focus on BBB‐penetrant statins (MMSE outcomes).	Statins may provide long‐term cognitive benefit in these patients who are indicated for lipid‐lowering treatment.	[71]
Lundin et al.; 2023	Hypertension control and combined use of antihypertensives with statins/metformin associated with reduced progression from MCI to dementia.	Evaluate whether the use of antihypertensive drugs reduces the risk of AD in patients with MCI, with a similar effect across classes, and may be enhanced when combined with statins or metformin.	The use of antihypertensive drugs reduces the risk of AD in patients with MCI, with similar effects across classes, and may be enhanced when combined with statins or metformin.	[77]
Joseph et al.; 2023	The PREVENTABLE study, a randomized, double‐blind clinical trial, evaluates whether controlling vascular risk factors with statins can delay or reduce the onset of dementia, including AD, especially its vascular or mixed forms, which are very common in the elderly.	Evaluate whether high‐intensity statin therapy prevents dementia and prolongs disability‐free survival in older adults.	Possible reduction or delay of dementia onset via improved cerebrovascular health and reduced vascular risk factors.	[81]
Wei et al.; 2022	Randomized, double‐blind, placebo‐controlled trial of simvastatin (40 mg/day) vs. placebo for 12 weeks in hyperlipidemic patients; plasma Aβ transport biomarkers measured.	Modulation of lipid metabolism, BBB function, and Aβ transport/clearance pathways.	Modulation of plasma Aβ transport with potential reduction of brain Aβ deposition.	[67]
Zheng et al.; 2025.	To evaluate whether statin use (lipid‐lowering drugs commonly prescribed to lower cholesterol) is associated with changes in brain structure in older people, including those with mild cognitive impairment or AD	Statins modulate lipid metabolism affecting Aβ and tau pathways; effects influenced by APOE ε4, age, and cardiovascular comorbidities.	Associated with increased cortical thickness and possible slowing of neurodegeneration in early AD/MCI; associative, not causal effect.	[66]
Grau‐Jurado et al.; 2025	Observational cohort analysis evaluating associations between commonly used medications (e.g., statins and metformin) and cognitive decline in AD.	The study examined how comorbidity‐related medications may influence cognitive decline and disease progression in AD.	No specific drug directly slows AD progression; some comorbidity medications show statistical associations with cognitive outcomes. Findings may help identify therapies influencing cognition and guide future targeted clinical trials.	[73]
Nabizadeh et al.; 2023	This study evaluates whether chronic statin use influences brain Aβ deposition and metabolism using PET imaging. Includes healthy controls, MCI, and AD patients. Observational analysis of long‐term statin exposure rather than experimental treatment.	The study links AD pathology to Aβ deposition, synaptic degeneration, metabolic dysfunction, and vascular–metabolic factors.	Statin use was linked to lower amyloid deposition in cognitively normal individuals, but not to slower progression in established AD.	[65]
Park et al.; 2025	This evaluates the impact of statin use on mortality and disease progression in patients with AD. Statins investigated for potential neuroprotective effects and modulation of vascular risk factors involved in AD pathogenesis.	Statin use was associated with reduced mortality in AD patients, without clear evidence of slowed cognitive progression.	Statin use associated with reduced all‐cause mortality in patients with AD, particularly in prior statin users. No significant effect observed on clinical disease progression, assessed by time to memantine initiation.	[70]
Nedelec et al.; 2023	Retrospective observational analysis of primary care and UK Biobank data comparing early clinical features of AD, PD, and DLB up to 15 years before diagnosis. Identifies symptoms, comorbidities, and treatments associated with future AD development.	The study identified early clinical prodromes and inflammatory pathways involving NF‐κB, p38 MAPK, and COX‐2 that may contribute to AD neurodegeneration.	No direct evaluation of therapeutic interventions. Identifies early symptoms and conditions preceding AD diagnosis. Suggests early management of systemic factors (e.g., depression, metabolic or inflammatory disorders, and sleep disturbances) may influence future disease trajectories.	[82]
Jeong et al.; 2021	Large observational cohort study using the Korean Health Insurance Database to assess the association between statin use and AD incidence in adults ≥ 60 years. Evaluates duration of use and cumulative statin dose as exposure variables.	Statins may modulate AD‐related pathways through lipid‐lowering, anti‐inflammatory, and antioxidant effects, including NF‐κB/NLRP3 signaling.	No overall association between statin use and AD risk. Dose–response relationship observed: short‐term or low cumulative use associated with higher AD risk, whereas long‐term and persistent use associated with reduced AD risk. Suggests inverse J‐shaped relationship between statin exposure and AD incidence.	[60]
Rajan et al.; 2024	This observational cohort study evaluated the initiation of statin therapy in older adults (mean age ≈72 years). Participants were classified based on statin use during follow‐up. Outcomes were compared between individuals who initiated statins and those who had never used them.	Statins may influence AD risk by lowering LDL cholesterol, improving vascular function, and modulating inflammation, with possible interactions involving APOE ε4, lipid metabolism, and Aβ accumulation.	Statin initiation was associated with lower AD risk and slower cognitive decline, particularly among APOE ε4 carriers.	[61]
Murphy et al.; 2023	This study evaluated chronic statin use (e.g., atorvastatin and simvastatin) in older adults with mild to moderate AD. It assessed the real‐world association between ongoing statin therapy, cognitive decline, dementia progression, and adverse events.	Statins may indirectly affect AD‐related pathways through cholesterol metabolism, anti‐inflammatory activity, and vascular effects, although their impact in established AD appears limited.	Continuous statin use was not associated with a significant slowing of cognitive decline in patients with mild to moderate AD, as measured by standard scales (ADAS‐Cog, CDR‐Sb, DAD). Although statins appeared safe in this population, they did not significantly modify the cognitive or clinical progression of the disease.	[69]
Kim et al.; 2021	This nested case–control study analyzed national cohort data from Korea to examine the association between previous statin use and AD. The study compared 17 172 patients with AD with 68 688 matched healthy controls (age, sex, income, and residence). Statin exposure referred to prior use of HMG‐CoA reductase inhibitors for cholesterol reduction.	Statins may lower AD risk through anti‐inflammatory effects, modulation of cholesterol/Aβ pathways, and improved vascular function, although mechanisms were not directly assessed.	Statistical analysis showed that longer statin use was associated with a slightly lower risk of developing AD. The adjusted odds ratio was 0.95 (95% CI 0.92–0.98; *p* = 0.003), indicating a statistically significant reduction in risk.	[62]
Lee et al.; 2025	This retrospective observational study analyzed clinical data from 11 university hospitals using a Common Data Model. It examined the association between LDL cholesterol levels and the risk of incident dementia, including AD‐related dementia. Participants were classified by LDL‐C thresholds, and statin use was also evaluated as a potential influencing factor.	The study links LDL‐C levels to dementia risk through vascular, metabolic, and inflammatory mechanisms relevant to AD.	The study did not test a specific experimental treatment, but it did examine how: Lower LDL cholesterol levels and statin use are associated with a lower risk of developing dementia, including AD.	[63]
Zimmerman et al.; 2025	This study evaluated the initiation of statin therapy using a target trial emulation design based on observational data from over 700 000 adults in a large healthcare system. Statins, commonly used to reduce LDL cholesterol and cardiovascular risk, were examined for their potential influence on dementia incidence, given the link between hypercholesterolemia, vascular damage, inflammation, and AD‐related processes.	Vascular and metabolic mechanisms may contribute to AD, but in this real‐world study statin‐related effects did not translate into reduced AD incidence.	During the first year after statin initiation, diagnoses of AD or related dementia were more frequent, likely reflecting increased healthcare contact and earlier detection. However, after the first year of follow‐up, statin use was not significantly associated with either an increased or reduced risk of dementia.	[59]
Ye et al.; 2025	This is a longitudinal observational study based on data from the UK Biobank. Approximately 371 019 individuals without dementia at baseline were followed for approximately 9 years. The aim of the study was to evaluate the association between statin use and the risk of developing AD and to understand how this association could vary based on genetic and personal factors (e.g., APOE ε4 allele, age, sex, and heart disease).	The study examined links between cholesterol metabolism, genetic susceptibility, and AD risk, including APOE ε4, polygenic risk, and clinical factors.	Statin use has been associated with a higher risk of developing AD compared to non‐users.	[75]
Collin et al.; 2022	The study looks at the association between statin (cholesterol‐lowering drug) use and CoQ10 supplements and how they may influence changes in cognitive function and potential outcomes in AD.	The article discusses how statins and CoQ10 may influence AD‐related oxidative stress, mitochondrial function, lipid metabolism, and inflammation.	The authors observed potential associations between statin use and changes in cognitive scores over time, along with possible benefits of CoQ10 related to oxidative stress. However, the findings suggest biological correlations rather than strong clinical evidence of therapeutic efficacy.	[78]
Royall et al.; 2024	Researchers analyzed data from 1725 participants from the ADNI cohort, examining statin use and the presence of the APOE ε4 allele. The study evaluated whether statins moderated the relationship between APOE ε4, C‐reactive protein (CRP), and dementia severity.	The study examined two key biological pathways related to AD: genetic risk associated with the APOE ε4 allele and systemic inflammation measured by C‐reactive protein (CRP). Both factors are linked to increased risk of cognitive decline and dementia progression.	Statin use was associated with less severe dementia in individuals carrying the APOE ε4 allele compared with non‐users. In contrast, no significant effect of statins on dementia severity was observed in participants without the APOE ε4 allele.	[76]
Lee et al.; 2021	The study performed bioinformatic analyses of transcriptomic data rather than a clinical trial. Gene expression in blood from AD patients was compared with that of individuals treated with statins using public datasets. Additional analyses examined gene expression changes in mouse neural stem cells exposed to statins to explore related biological pathways.	The analysis identified gene expression changes linking AD and statin‐related pathways. Several genes dysregulated in AD showed opposite expression patterns in individuals treated with statins, suggesting that statins may modulate some biological pathways altered in the disease.	The gene profile associated with AD includes pathways such as those of the ribosome, spliceosome, and intracellular transport. Statins appear to modulate the expression of genes involved in key cellular functions, including immune response pathways and lipid transport.	[68]
Mundal et al.; 2022	This prospective population‐based cohort study compared 3520 individuals with familial hypercholesterolemia (FH) with 69 713 age‐ and sex‐matched controls. It evaluated the observational exposure to long‐term statin use, drugs that lower LDL cholesterol and influence vascular inflammatory processes.	Although statins may theoretically affect neuroinflammation, oxidative stress, and protein accumulation, this observational study found no significant association with dementia risk.	No significant increase in total dementia risk was observed in patients with familial hypercholesterolemia compared with controls. Similarly, no association was found between FH or cumulative statin use and the risk of AD or related dementia. Overall, statins showed neither a clear protective nor harmful effect.	[74]
Ge et al.; 2024	The authors evaluated whether the use of statins—drugs commonly prescribed to lower cholesterol—was associated with variations in the risk of developing dementia, including AD, in a population of older Japanese people.	The study delves into the biological pathways related to dementia and lipid metabolism (cholesterol and statins), exploring how pharmacological intervention on these pathways may have a protective or associative effect on the onset of the disease.	An association between statin use and the risk of dementia (including AD) has been observed, suggesting that use of these drugs may influence the risk of developing the disease in older adults, although this study does not prove a direct causal effect	[64]
Degrush et al.; 2022	The study evaluated the combined effect of simvastatin, L‐arginine, and tetrahydrobiopterin (BH4) in individuals with mild AD or mild cognitive impairment. These agents enhance the endothelial nitric oxide (eNOS) pathway, aiming to improve cerebral blood flow. The primary outcome was change in cerebral blood flow measured by MRI, with cognitive function as a secondary outcome.	The study targeted the eNOS/NO pathway using simvastatin, L‐arginine, and BH4 to improve cerebral blood flow in AD‐related vascular dysfunction.	Treatment was associated with a significant increase in cerebral blood flow, with approximately +13% in the limbic system and +15% in the cerebral cortex. Cognitive scores also showed modest improvement, with MMSE increasing from about 24.2 to 26.0 after 16 weeks. Greater cognitive improvement was observed in participants with the largest increases in cerebral blood flow.	[79]
Petek et al.; 2025	This cross‐sectional observational study analyzed 3074 patients with AD using data from the Swedish SveDem registry and laboratory records. It examined statin use (mainly simvastatin and atorvastatin) at the time of AD diagnosis rather than testing an experimental treatment.	The study examined lipid profiles, statin use, and MMSE performance, highlighting lipid metabolism as a potential contributor to AD‐related processes.	Statin use was more common in younger patients and in those with cardiovascular comorbidities. Simvastatin use was associated with slightly higher MMSE scores at the time of AD diagnosis compared with nonusers. Among patients not taking statins, higher LDL‐C, total cholesterol, and HDL‐C levels were correlated with better cognitive scores, an association not observed in statin users.	[72]
Usefi et al.; 2024	The study compared rosuvastatin, silymarin, and placebo in patients with AD and dyslipidemia within a historical cohort. It evaluated biological markers including serum total tau levels and the expression of miR‐124‐3p, a microRNA involved in neuronal metabolism and neuroprotection.	The study examined serum tau and miR‐124‐3p as AD‐related markers, linking reduced tau and increased miR‐124‐3p to potential neuroprotection and slower progression.	The findings suggest possible effects through lipid metabolism and inflammatory modulation by rosuvastatin. Increased expression of miR‐124‐3p may regulate genes involved in neuronal homeostasis, oxidative stress response, and synaptic plasticity. The reduction in tau levels also indicates a potential attenuation of tau‐related neurodegenerative processes.	[80]

## 4 Epidemiology, Pathophysiology, and Clinical Manifestations in PD

PD is a leading cause of disability and the second most common neurodegenerative disorder, affecting approximately 0.5%–1% of old people over 60 years worldwide [83]. Given the progressive aging of the population, the number of affected people is estimated to double in the next 20 years [84].

Neuropathologically, AD is characterized by a progressive degeneration of dopaminergic neurons located in the pars compacta of the substantia nigra, a structure in the midbrain [85]. This degeneration is associated with the presence of Lewy bodies, cytoplasmic inclusions primarily composed of insoluble aggregates of α‐synuclein [86]. However, PD is not confined to this region; it also involves multiple areas of the CNS, including non‐dopaminergic neuronal populations. Beyond the loss of dopaminergic neurons in the substantia nigra pars compacta, PD is increasingly recognized as a multisystem neurodegenerative disorder affecting numerous structures of the CNS [87]. According to Braak's hypothesis, the pathological process spreads in a caudo‐rostral pattern, initially involving the olfactory bulb and brainstem nuclei, particularly the dorsal motor nucleus of the vagus, before extending to the midbrain, the limbic system, and ultimately the neocortex [88]. This anatomical progression is consistent with the early appearance of non‐motor symptoms, including hyposmia, REM sleep behavior disorder, autonomic dysfunction, and mood disturbances, which frequently precede the onset of classical motor manifestations by several years [89]. Neurochemically, in addition to the nigrostriatal dopaminergic deficit, noradrenergic (locus coeruleus), serotonergic (raphe nuclei), and cholinergic (basal nucleus of Meynert) systems are also involved [90, 91]. This multiple degeneration contributes to the complexity of the clinical phenotype, explaining the presence of cognitive, affective, and autonomic symptoms in advanced stages of the disease [90, 91].

Finally, at the cellular and molecular levels, neurodegeneration is associated with mitochondrial dysfunction, oxidative stress, microglia‐mediated neuroinflammation, and dysregulation of iron metabolism in the substantia nigra [92, 93]. Moreover, interaction between genetic and environmental factors is thought to modulate these pathogenic processes, contributing to the selective vulnerability of specific neuronal populations [94]. Clinical diagnosis is primarily based on the presence of motor symptoms, such as resting tremor, bradykinesia, and muscle rigidity, and non‐motor manifestations, including anosmia, constipation, depression, and sleep and behavioral disturbances [95]. As the disease progresses, additional clinical signs may appear, such as pain, altered autonomic function, and cognitive impairment [96]. To improve the diagnostic accuracy of PD, the International and Movement Disorder Society (MDS) has proposed a set of criteria based on a specialized clinical neurological examination demonstrating a Parkinsonian syndrome defined by the presence of bradykinesia and at least one other cardinal motor feature (rigidity or tremor at rest) [97].

The MDS criteria operationalize two levels of diagnostic certainty for the disease: clinically certain and clinically probable. The first category establishes a series of criteria aimed at maximizing specificity at the expense of sensitivity, while the criteria for the second level aim for greater sensitivity [98]. Current therapeutic strategies for PD are primarily symptomatic and aim to improve motor and non‐motor manifestations without altering the underlying neurodegenerative process. Pharmacological treatments mainly focus on restoring dopaminergic transmission, with levodopa representing the most effective and widely used therapy, often administered in combination with peripheral dopa decarboxylase inhibitors [99]. Other commonly employed medications include dopamine agonists, monoamine oxidase‐B (MAO‐B) inhibitors, and catechol‐O‐methyltransferase (COMT) inhibitors, which help prolong dopamine availability in the brain [100]. In selected patients, advanced therapeutic approaches such as deep brain stimulation may also be considered to manage motor complications [101]. Despite these options, no currently available treatment is capable of halting or reversing disease progression, and PD remains an incurable neurodegenerative disorder.

**FIGURE 2:**
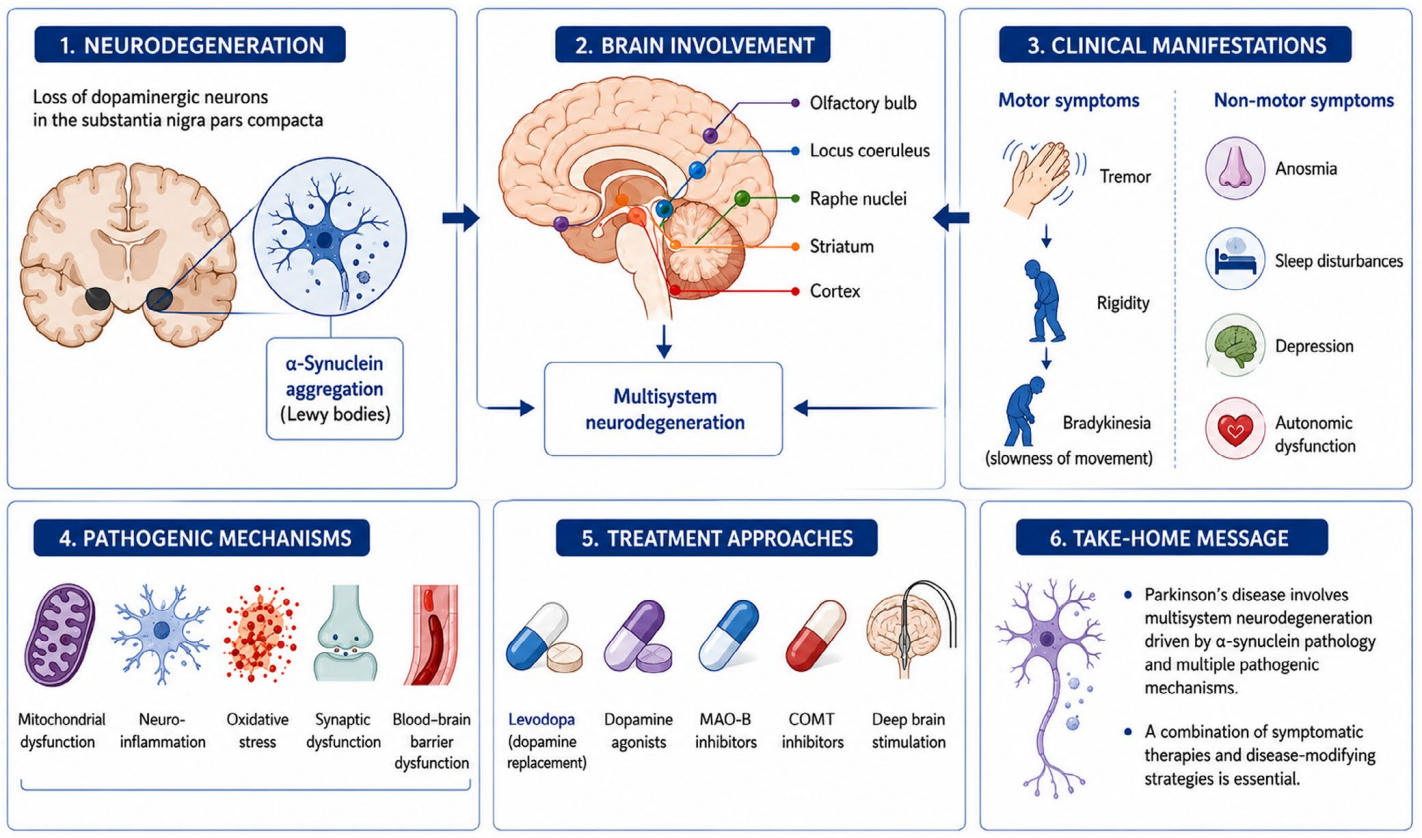
Nigrostriatal circuit dysfunction, motor, and non‐motor symptoms as well as pharmacological treatments in PD.

As stated, recent years have seen growing interest in drug repurposing strategies aimed at identifying compounds with potential disease‐modifying properties. Among these, statins have attracted considerable interest due to their pleiotropic effects, including anti‐inflammatory, antioxidant, and neuroprotective actions. The following sections summarize the current evidence on the role of statins in PD, beginning with findings from preclinical models and subsequently reviewing data from clinical studies. A graphical overview of PD is provided in Figure 2.

### 4.1 Preclinical Studies Investigating Statins in PD From 2021 to 2025

Growing evidence from experimental models suggests that statins may influence several molecular processes implicated in PD, including neuroinflammation, mitochondrial dysfunction, and α‐synuclein pathology. In a rat model of MPP^+^‐induced nigrostriatal injury, simvastatin pretreatment reduced dopaminergic damage, oxidative stress, and inflammatory protein expression within the substantia nigra and striatum, indicating that modulation of the neuroinflammatory response contributes to its protective effects [102]. Consistent with these findings, simvastatin prevented dopaminergic neuron loss and improved motor behavior in the MPTP mouse model of PD by inhibiting the formation of neurotoxic A1 reactive astrocytes and limiting astrocyte‐mediated neuronal death, as suggested by Du and Bu in their work [103].

Statins may also modulate pathological protein aggregation, a key hallmark of PD. In cellular models of synucleinopathy, lovastatin has been shown to reduce α‐synuclein phosphorylation and aggregation induced by preformed fibrils, while also attenuating oxidative stress and inhibiting casein kinase 2 activity [104]. In addition to these effects, statins appear to regulate mitochondrial quality control mechanisms. In the study of Liu et al. [45], lovastatin has been shown to enhance mitophagy through SHP2‐mediated activation of Parkin‐dependent mitochondrial clearance, thereby promoting the removal of damaged mitochondria and alleviating parkinsonian phenotypes in experimental models. Beyond neurodegeneration, statins may also influence mechanisms underlying treatment‐related complications. In a rat model of L‐DOPA‐induced dyskinesia, simvastatin reduced dyskinetic responses and inflammatory signaling through interactions with angiotensin II/AT1 receptors, cholesterol metabolism, and ROCK pathways [105]. Furthermore, emerging evidence suggests that advanced drug delivery strategies may enhance the therapeutic potential of statins in neurodegenerative disorders. For instance, rosuvastatin‐loaded nanoemulsions have been shown to reduce oxidative stress and glial activation while protecting neuronal tissue in experimental models of neuroinflammation, highlighting the potential of nanotechnology‐based approaches to improve brain delivery of statins [106]. Preclinical studies investigating the use of statins for the treatment of PD between 2021 and 2025 are summarized in Table 3.

**TABLE 3:** Summary of preclinical studies evaluating statins as a pharmacological intervention in PD from 2021 to 2025.

First author and year of publication	Intervention	Key findings–related pathway	Effect on PD	References
Rubio‐Osornio et al.; 2023	Simvastatin has anti‐inflammatory and antioxidant properties and modulates microglial activity, mechanisms relevant to PD. In the MPP^+^‐induced PD model, its effects on neuroinflammation, oxidative stress, and dopaminergic neuron survival were evaluated.	Simvastatin may support dopaminergic neuron survival by reducing neuroinflammation and oxidative stress, key mechanisms in PD neurodegeneration.	The study suggests that simvastatin may have a neuroprotective effect in PD, acting primarily on neuroinflammation, oxidative stress, and dopaminergic neuron survival rather than directly on α‐synuclein aggregation.	[102]
Du & Bu; 2021	The study evaluates whether simvastatin protects dopaminergic neurons in the MPTP mouse model of PD and investigates if this neuroprotective effect is mediated by inhibition of A1 reactive astrocytes.	Simvastatin exerts a neuroprotective effect in the MPTP model of PD by reducing dopaminergic neuron loss, likely through inhibition of A1 reactive astrocytes and the resulting neuroinflammatory neurotoxicity.	Simvastatin may exert neuroprotective effects in PD models by preventing dopaminergic neuron degeneration, likely through inhibition of A1 reactive astrocyte activation and the associated neuroinflammation.	[103]
Dai et al.; 2021	The study evaluates whether lovastatin can modulate key pathogenic processes in PD, including α‐synuclein aggregation and phosphorylation, oxidative stress, and kinase activation.	Lovastatin targets several PD‐related pathways, including α‐synuclein aggregation and phosphorylation (S129), oxidative stress, kinase activation (e.g., CK2), and epigenetic regulation of genes involved in neuronal survival.	Lovastatin reduces α‐synuclein aggregation and S129 phosphorylation, inhibits CK2 activity, decreases oxidative stress markers, and modulates histone acetylation (H3/H4), suggesting multiple neuroprotective mechanisms in PD models.	[104]
Lopez‐Lopez et al.; 2023	The study evaluated statins, AT1 receptor antagonists, and ROCK inhibitors in L‐DOPA‐treated PD models to assess their effects on L‐DOPA‐induced dyskinesia. The combined modulation of cholesterol metabolism and inflammatory pathways was investigated.	The study links AT1 receptor, cholesterol, and ROCK pathways to PD‐related inflammation, oxidative stress, dopaminergic dysfunction, and L‐DOPA‐induced dyskinesia.	In animal models, treatment reduced L‐DOPA‐induced dyskinesia and involuntary movements, improving therapeutic response. The drugs also attenuated neuroinflammation and dopaminergic neurodegeneration.	[105]
Saberi et al.; 2025	The study investigated rosuvastatin‐loaded nanoemulsions in an animal model to evaluate their effects on neuroinflammation and oxidative stress in neurodegenerative diseases.	The formulation targeted two key pathological pathways: neuroinflammation and oxidative stress. Treatment reduced astrocyte activation and lipid peroxidation while increasing brain sulfhydryl levels, indicating antioxidant effects.	The study does not directly investigate PD or AD, but targets shared pathogenic mechanisms such as neuroinflammation and oxidative stress. These effects may reduce dopaminergic degeneration in PD and neuronal damage associated with Aβ and tau in AD.	[106]

### 4.2 Clinical Evidence Linking Statin Use to PD

Several observational and clinical studies have investigated the potential relationship between statin use and PD, although the findings remain heterogeneous and sometimes conflicting. Some studies suggest a possible beneficial role of statins on disease progression. For instance, Palermo et al. [107] reported that patients with newly diagnosed PD who were long‐term statin users showed a slower progression of motor symptoms over a 4‐year follow‐up period compared with nonusers, suggesting a potential disease‐modifying effect on motor decline.

Conversely, other investigations have raised concerns regarding a possible association between statin exposure and an increased risk of PD. In their paper, Kim and colleagues [108] found that long‐term use of lipophilic statins was associated with a higher risk of developing PD. However, the authors emphasized that this association does not demonstrate causality and may reflect residual confounding factors or biases inherent to observational designs.

Other studies have reported more nuanced results. Nguyen et al. [109] did not observe a significant association between overall statin use and PD incidence; however, the use of lipophilic statins was associated with a statistically significant reduction in PD risk among women in their cohort, with evidence of a possible dose–response relationship. These observations suggest that certain statins may exert neuroprotective effects, potentially through anti‐inflammatory and antioxidant mechanisms or modulation of metabolic pathways involved in neurodegeneration; although further experimental validation is required.

Similarly, Ge et al. [110] reported an inverse association between cumulative statin exposure and PD risk in an elderly Japanese population, with greater cumulative use associated with a lower incidence of the disease. Nevertheless, the authors emphasized that prospective studies and randomized clinical trials are needed to determine whether this association reflects a causal protective effect.

In contrast, other evidence points toward a potentially unfavorable impact of statins on disease progression. Jeong and colleagues [111] observed that statin use was associated with a more pronounced decline in dopamine transporter levels in brain regions involved in motor and cognitive functions. In the same study, statin users required more rapid escalation of levodopa dosage and showed a higher likelihood of conversion to dementia, suggesting a possible negative influence on the clinical course of PD.

Large observational analyses have also examined the broader relationship between pharmacological treatments and disease onset. For example, Chang et al. [112] identified statistical associations between several medications, including statins, and the onset of PD. However, the authors emphasized that these findings should be interpreted as hypothesis‐generating rather than evidence of direct biological causality. Interventional studies investigating statins as potential disease‐modifying therapies have produced mixed results. In a randomized clinical trial, Lin et al. [113] evaluated lovastatin as a potential treatment for early‐stage PD. Although the study did not demonstrate definitive evidence of disease slowing, a trend toward clinical benefit was observed, suggesting that further research into statin‐related pathways, such as α‐synuclein aggregation, oxidative stress, inflammation, and lipid metabolism, may be warranted. More recently, Malatt et al. [114] reported that statin use was associated with a later age at onset of PD symptoms, although the study did not demonstrate a curative or definitive disease‐modifying effect. The proposed mechanisms underlying this association, including modulation of inflammatory pathways, sympathetic activity, and cellular metabolism, remain speculative and require confirmation through targeted experimental and clinical studies.

Conversely, the randomized trial conducted by Stevens et al. [115] did not support a disease‐modifying role for simvastatin in PD. Using widely accepted clinical endpoints, including motor progression scales, the study provided robust evidence that simvastatin does not significantly alter disease progression [115]. These findings reinforce the need to identify more specific biological targets for disease‐modifying therapies, such as pathways related to α‐synuclein aggregation or neurotrophic signaling. In addition, epidemiological data from Oveisgharan et al. [116] indicated that older adults taking statins had a lower risk of developing parkinsonism compared with non‐users. The authors suggested that this association may partly reflect the beneficial effects of statins on cerebral vascular health, particularly through the reduction of cerebral atherosclerosis, highlighting the potential interaction between vascular factors and Parkinsonian manifestations [116]. Genetic studies have further complicated the interpretation of the relationship between lipid metabolism and PD. Using Mendelian randomization approaches, Huang et al. [117] reported that genetic inhibition of PCSK9 was associated with an increased risk of PD. Similarly, genetic inhibition of HMGCR, the pharmacological target of statins, was also linked to a higher risk of PD, suggesting that alterations in lipid metabolism may influence the vulnerability of dopaminergic neurons [117]. Notably, the study further indicated that PCSK9 modulation may exert disease‐specific effects across neurodegenerative disorders, potentially increasing risk in PD while showing neutral or protective associations in other conditions [117]. Recent clinical evidence has begun to clarify the potential role of statins in PD risk and progression. A large‐scale systematic review and meta‐analysis by Mady et al. [118], including over 4 million individuals, reported that statin use is associated with a modest reduction in PD risk (RR ≈0.86), suggesting a possible neuroprotective effect in preclinical stages. Notably, lipophilic statins appeared to confer greater benefit, likely due to enhanced blood–brain barrier penetration. However, no consistent effects were observed on disease progression or motor outcomes, highlighting a discrepancy between preventive associations and therapeutic efficacy in established PD. These findings suggest that statins may primarily influence early pathogenic processes rather than modifying disease course once neurodegeneration is established. Clinical studies evaluating the use of statins for the treatment of PD between 2021 and 2025 are summarized in Table 4.

**TABLE 4:** Summary of clinical studies evaluating statins as a pharmacological intervention in PD from 2021 to 2025.

First author and year of publication	Intervention	Key findings–related pathway	Effect on PD	References
Palermo et al.; 2021	The study compares PD patients taking long‐term statins with those not using statins to evaluate differences in motor symptom progression using clinical scales such as UPDRS III.	The study investigates whether long‐term statin use is associated with a slower progression of motor symptoms in PD.	Long‐term statin use may be associated with slower progression of rigidity and some motor symptoms in de novo PD patients.	[107]
Kim et al.; 2022	The study investigates the association between statin use and the risk of developing PD in Korean patients with hyperlipidemia using a nested case–control design based on a national database.	PD is characterized by progressive loss of dopaminergic neurons in the substantia nigra and intraneuronal aggregation of α‐synuclein (Lewy bodies), which underlie the main motor and non‐motor symptoms of the disease.	Statin use for ≥ 12 months was associated with a higher risk of developing PD compared with < 6 months of use, an effect observed only with lipophilic statins; although statins have anti‐inflammatory and pleiotropic effects, their role in neurodegeneration remains controversial.	[108]
Nguyen et al.; 2023	A prospective cohort study from the E3N cohort evaluating the association between statin use and PD incidence in women, using exposure models with a 5‐year lag to reduce reverse causality.	Lipophilic statins may affect PD‐related neuroinflammation, oxidative stress, and lipid signaling, although observational data do not prove causality.	Overall statin use was not significantly associated with PD incidence, but lipophilic statin use ≥ 5 years earlier was linked to a lower PD risk (HR ≈0.70), with some cumulative dose categories showing a significant inverse association.	[109]
Ge et al.; 2024	A nested case–control observational study using Japanese healthcare data examining the association between statin use, cumulative statin exposure, and the risk of developing PD.	Possible mechanisms include reduced neuroinflammation and microglial activation, support of dopaminergic neuron survival, attenuation of α‐synuclein aggregation, and improved cerebral vascular function.	Chronic use of high‐dose statins is statistically associated with a lower risk of developing PD in older adults. Because it is an observational study, it cannot demonstrate causality, only an association.	[110]
Jeong et al.; 2021	An observational clinical study evaluating whether statin use in PD patients is associated with dopaminergic decline and with motor and cognitive disease progression.	The study examined statin effects on the nigrostriatal dopaminergic system, focusing on neuroinflammation, lipid metabolism, and dopamine transport.	Statin use was associated with faster dopaminergic decline and worse motor/cognitive outcomes in PD, independently of cholesterol levels.	[111]
Chang et al.; 2021	The study analyzes a large cohort of Taiwanese patients with diabetes to evaluate whether treatment with pioglitazone and statins is associated with a reduced incidence of PD.	Statins and pioglitazone may protect against PD by reducing neuroinflammation and oxidative stress, with pioglitazone also supporting mitochondrial function via PPAR‐γ activation.	Pioglitazone was associated with a reduced incidence of PD in diabetic patients, and concomitant statin use appeared to enhance this protective effect, possibly through anti‐inflammatory, metabolic, and neuroprotective mechanisms.	[112]
Lin et al.; 2021	The trial evaluated lovastatin, a drug belonging to the statin class, to determine its potential neuroprotective effects in patients with early‐stage PD.	Lovastatin may exert neuroprotective effects in PD by reducing α‐synuclein aggregation, oxidative stress, and lipid‐related inflammation.	This randomized clinical trial explores the potential neuroprotective role of lovastatin in PD by targeting metabolic and inflammatory pathways, although no definitive clinical benefit was demonstrated and larger long‐term studies are needed.	[113]
Malatt et al.; 2025	A retrospective observational study evaluating whether prior use of adrenergic blockers, statins, and NSAIDs is associated with a later age at onset of PD motor symptoms.	Proposed mechanisms include reduced neuroinflammation, microglial activation, sympathetic signaling, and vascular‐metabolic dysfunction.	Adrenergic blockers, statins, and NSAIDs were associated with a significantly later age at onset of PD in multivariate analyses, while antidiabetic drugs and β‐agonists showed weaker similar trends.	[114]
Stevens et al.; 2022	A randomized controlled clinical trial designed to evaluate whether simvastatin has a disease‐modifying effect by slowing the progression of motor symptoms in PD.	The study is based on the hypothesis that the anti‐inflammatory, antioxidant, and potential neuroprotective effects of statins could slow neurodegenerative progression in PD.	Simvastatin did not show disease‐modifying effects in moderate PD, suggesting no clear clinical benefit on motor progression in this population.	[115]
Oveisgharan et al.; 2022	An observational longitudinal study evaluating whether statin use in older adults is associated with incident parkinsonism and its relationship with cerebral atherosclerosis.	The study suggests that statins may indirectly reduce the risk of parkinsonism by lowering cholesterol and decreasing cerebral atherosclerosis, a vascular factor associated with Parkinsonian motor symptoms.	Statin use was associated with a lower risk of developing parkinsonism in older adults, partly mediated by reduced cerebral atherosclerosis (~17%), suggesting additional non‐vascular mechanisms may also contribute.	[116]
Huang et al.; 2024	A Mendelian randomization study evaluating whether genetic variants associated with PCSK9 inhibition (a target of LDL‐lowering drugs) causally influence the risk of neurodegenerative diseases.	Genetic proxies for PCSK9 and HMGCR inhibition were used to explore links between LDL metabolism, neuroinflammation, protein aggregation, and neurodegeneration.	Genetic proxies of PCSK9 and HMGCR inhibition were associated with increased PD risk, suggesting a possible link between reduced cholesterol signaling and dopaminergic vulnerability.	[117]

## 5 Conclusions and Future Perspectives

AD and PD represent the most prevalent and challenging neurodegenerative disorders worldwide, with their rising incidence largely reflecting the progressive aging of the global population. Despite significant advances in elucidating their cellular and molecular mechanisms, effective disease‐modifying therapies remain limited. In this context, drug repurposing strategies have gained increasing attention, offering the potential to accelerate therapeutic development by leveraging compounds with well‐established pharmacological profiles and safety. Among these candidates, statins have emerged as particularly promising due to their pleiotropic biological effects beyond cholesterol lowering. Experimental and translational evidence suggests the ability of statins to modulate key pathogenic pathways involved in neurodegeneration, including neuroinflammation, oxidative stress, mitochondrial dysfunction, and protein aggregation of Aβ and α‐synuclein.

In accordance with the above, recent meta‐analyses have strengthened the evidence supporting a possible association between statin use and reduced risk of neurodegenerative diseases. In particular, Filho et al. [58] reported that statin use was associated with a significantly lower risk of all‐cause dementia and AD, with more pronounced effects in patients with type 2 diabetes, prolonged exposure, and specific statins such as rosuvastatin. Similarly, Mady et al. [118] found a reduced risk of PD among statin users, although current evidence did not support a significant disease‐modifying effect on PD progression. Notably, both studies highlighted important limitations related to the predominantly observational nature of the available clinical evidence and the potential influence of confounding factors. In this context, the integration of preclinical and clinical findings may help explain the discrepancy between the robust neuroprotective mechanisms observed experimentally and the heterogeneous outcomes reported in clinical studies. Experimental evidence consistently supports anti‐inflammatory, antioxidant, anti‐amyloid, and neuroprotective effects of statins, whereas clinical efficacy may depend on factors such as treatment timing, patient selection, statin pharmacological properties, and disease stage.

In line with these findings, our review showed that preclinical studies consistently support pleiotropic neuroprotective effects of statins, whereas clinical evidence remains more heterogeneous and less conclusive. This discrepancy may reflect differences in study design, disease stage, treatment duration, patient selection, and pharmacological characteristics of individual statins. Overall, the available evidence suggests that statins may exert biologically relevant neuroprotective effects, although their translation into consistent clinical benefit remains to be fully established.

Moreover, their effects on vascular and metabolic pathways may indirectly contribute to neuronal resilience and cognitive preservation. Despite that, clinical evidence remains heterogeneous and, in some cases, contradictory. In fact, while several epidemiological studies suggest a protective association between long‐term statin exposure and reduced risk of cognitive decline or neurodegenerative disease, other investigations report neutral or context‐dependent outcomes. These discrepancies may reflect differences in study design, statin class, treatment duration, patient characteristics, and genetic background. In particular, factors such as blood–brain barrier permeability, statin lipophilicity, and genetic susceptibility, especially APOE genotype, may critically influence therapeutic responses. Future research should therefore focus on well‐designed longitudinal studies and randomized controlled trials to better define the potential neuroprotective role of statins in both AD and PD.

Biomarker‐driven approaches, including neuroimaging and fluid biomarkers, may facilitate the identification of patient subgroups most likely to benefit from therapeutic interventions. Moreover, a more comprehensive understanding of the interplay between lipid metabolism, neuroinflammation, and neurodegeneration could provide valuable insights for the development of more targeted and mechanism‐based treatment strategies. Ultimately, the integration of pharmacological interventions with lifestyle and preventive approaches may represent a key strategy for addressing the growing global burden of neurodegenerative diseases.

## Author Contributions

**Luigi Maria Chiatto:** writing – original draft. **Rocco Salvatore Calabrò:** supervision. **Carmela Rifici:** supervision, methodology. **Giuseppe Carota:** methodology. **Lucia Buccarello:** writing – review and editing, supervision. **Alessio Ardizzone:** conceptualization, writing – original draft. **Giuseppe Caruso:** writing – review and editing, supervision. **Emanuela Esposito:** conceptualization.

## Funding

The authors have nothing to report.

## Conflicts of Interest

The authors declare no conflicts of interest.

## Data Availability

No data were produced since this is a review article.
